# Ultra-light photosensor collars to monitor Arctic lemming activity

**DOI:** 10.1186/s40317-022-00302-1

**Published:** 2022-10-11

**Authors:** David Bolduc, Dominique Fauteux, Éric Bharucha, Jean-Marie Trudeau, Pierre Legagneux

**Affiliations:** 1 Centre d’Études Nordiques, Université Laval, 1045, avenue de la Médecine, Québec, QC G1V 0A6 Canada; 2 Centre for Arctic Knowledge and Exploration, Canadian Museum of Nature, P.O. Box 3443 station D, Ottawa, ON K1P 6P4 Canada; 3 Sentinel North Technological Instrument Development Platform, Université Laval, 2375 rue de la Terrasse, Québec, QC G1V 0A6 Canada

**Keywords:** Light sensor, Modern ethology, *Lemmus trimucronatus*, *Dicrostonyx hudsoniu*s, *Dicrostonyx groenlandicus*, Subterranean, Predator refugia

## Abstract

**Background:**

Studying the anti-predatory behavior of mammals represents an important challenge, especially for fossorial small mammals that hide in burrows. In the Arctic, such behaviors are critical to the survival of lemmings considering that predation risks are high every summer. Because detailed information about how lemmings use burrows as hideouts is still lacking, we developed a 1.59 g photosensitive collar to record any event of a small mammal moving between a dark area (e.g., burrow) and a bright area (e.g., outside the burrow). Tests of how collars affected lemming behavior were conducted in captivity in Cambridge Bay, Nunavut, Canada, in November 2019 and field tests were conducted on Bylot Island, Nunavut, Canada, in August 2021.

**Results:**

The device was made of two chemical batteries and a printed circuit board (PCB) equipped with a photosensor and a real-time clock that recorded amplitude transient thresholds of light (lux) continuously. In accordance with ethical use of such devices, we verified that no abnormal loss of body mass was observed in captive or free-ranging lemmings, and no difference in recapture rates were observed between those with and without a collar, though we could not test this for periods longer than 108 h. Measurements of light intensities revealed consistent patterns with high lux levels at mid-day and lowest during the night. Lemmings showed clearly defined behavioral patterns alternating between periods outside and inside burrows. Despite 24-h daylight in the middle of the summer, August nighttime (i.e., 11 PM to 4 AM) lux levels were insufficient for amplitude transient thresholds to be reached.

**Conclusion:**

By taking advantage of the long periods of daylight in the Arctic, such technology is very promising as it sets new bases for passive recording of behavioral parameters and builds on the prospect of further miniaturization of batteries and PCBs.

## Background

Lemmings are small burrowing rodents that are considered as keystone species in the Arctic tundra ecosystem. They represent the main prey of many avian and mammalian predators and are well-known for their 3 to 5 years high amplitude abundance cycles that have substantial impact on the local vertebrate diversity [1–5]. Identifying the causal factors of these cycles epitomizes one of the oldest ecological questions [6], and several hypotheses have been proposed such as regulation by food, predators or intrinsic factors, such as stress [7–9]. Recent studies conducted in the High-Arctic, provide compelling evidence that predators are an important factor behind this century-old enigma [10–13]. However, little is known about potential behavioral strategies that lemmings employ to face such heavy predation that peaks in summer during the presence of migratory predators.

A key aspect in predator–prey interactions is how predation shape the use of refuges by herbivores such as burrows, and vice versa [14]. Many fossorial herbivores like lemmings rely partly on roots in their diet [15], which allows them to browse in the safety of burrows. However, roots rarely consist of a sufficient food source especially in the summer characterized with numerous fine roots that are poorly nutritive [16, 17], and herbivores must make compromises between predator avoidance and food acquisition. Searching for mates and natal and breeding dispersal also force herbivores to move outside burrows [18]. Such behaviors can vary among individuals, especially between mate-searching polygamous males and nursing females, whose survival have different impacts on population growth. Unfortunately, lemming behavior is difficult to monitor through conventional methods, such as direct observations, because they use extensive networks of runways and tunnels to move around and are easy to lose sight of.

To better understand daily routines of lemmings during summer when they are highly exposed to both avian and mammalian predation [10, 19], we developed a new miniature photosensitive collar of 1.59 g that continuously records all transitions between dark and bright environments. We first assessed the physiological response of lemmings to these collars measured as body mass variations in captive and free-ranging lemmings over 24–72 h, and if lemmings with or without collars had different recapture rates, reflecting potential short-term impact on survival or behavior. The collar was designed to (i) work under Arctic summer conditions for a fossorial small mammal (i.e., temperatures range from − 5 °C to + 20 °C, high humidity and dirt); (ii) be resistant to tearing by claws; (iii) record light transitions continuously for > 2 weeks; and (iv) be reusable on other individuals when battery levels allow it. The recorded transitions between bright and dark environments would provide a reliable proxy of a lemming moving out of its burrow to open tundra, and vice versa, yielding information on the use of refuges. This device was developed considering the 24-h daylight during summer in the High-Arctic that creates ideal conditions for highly contrasting light intensities, which facilitates detection of transitions by the collars.

## Methods

### Development of the printed circuit board (PCB), reading hardware and software

A PCB was designed to hold the required components and compose the circuit mechanisms of the collar. Because the PCB was intended to be installed on a collar, it had to be flexible. This was achieved by first forming a 4-layer PCB circuit board fabricated with polyimide substrates. Although the final flexibility was somewhat limited by the rigid components installed on board, we reserved ‘keepout’ areas without components to allow specific bends that would fit with the round shape of the collar.

**Fig. 1:**
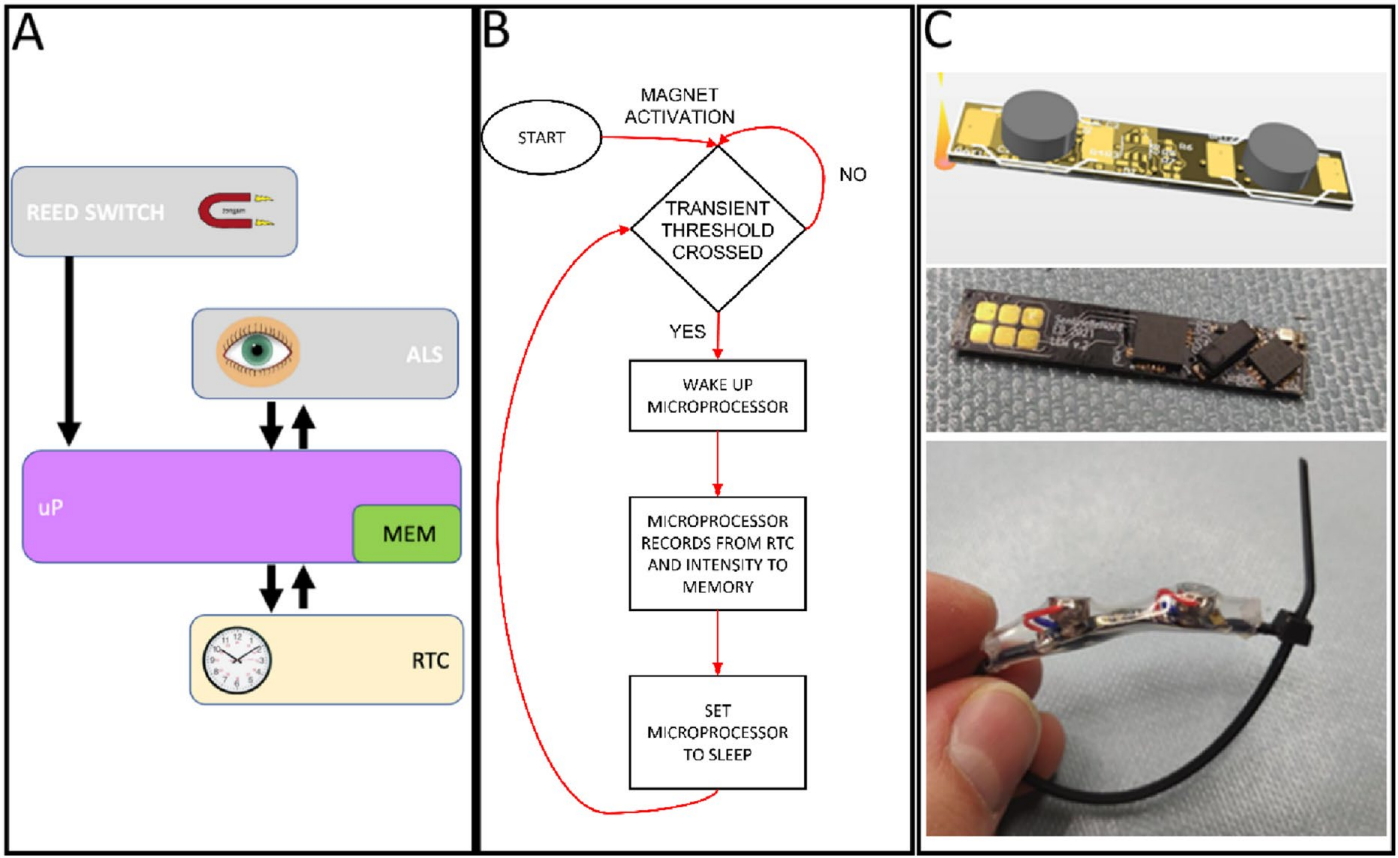
In **A** architecture of the instrumented lemming collar. The principal components are: the microprocessor (uP), with an ferroelectric memory (MEM); the ambient light sensor (ALS); the Real Time Clock (RTC) and a triggerable magnetic reed switch use to activate the device. In **B** algorithm of the light-sensitive collar. Intensity changes lead to analysis by the microprocessor only in situations when light level thresholds determined a priori are crossed. In **C** 3-D model (top), PCB assembly (center) and final device prototype (bottom)

The device was centered around a microcontroller, Texas Instruments MSP430FR2355, a real-time clock (RTC) AB0815 from Abracon with a quartz crystal reference, and an ambient light sensor LTR-308 ALS Lite-On corporation. Light intensities measured by the optic sensor were proportional to ambient light (luxes). The custom-made PCB with all components weighs 282 mg. When the two lightweight chemical batteries (330 mg each; Energizer® Zinc Air [Zn/O2]) were fixed on the PCB, the total mass was 942 mg. The board dimensions were 27.9 × 5.3 mm and the PCB was a flex board with a thickness of 200 µm. The RTC upkeeps the time and date while sustained by diminutive currents in the µA range. In addition, the microprocessor, a part of the microcontroller, also remained in a dormant mode to reduce battery consumption. The general architecture design is given in Fig. 1A.

Exits and entries from the burrow were detected and monitored by keeping track of ambient light transitions. Abrupt changes in light intensity create an interrupt signal that triggers further analysis by the microcontroller. After each light transition (e.g., from dark to light) and if the new state is maintained for at least 4 s, the transient amplitude (i.e., light intensity right after crossing a transient threshold), real time (from 00:00 to 23:59), and date of the event is stored in random access memory (RAM). Fleeting events that occurred within 4 s are ignored because they are generally assumed to be noise events such as passing under an object or in a small, illuminated portion of a tunnel. Very slow transitions are also ignored because they can be too easily triggered from changing weather (e.g., overcast vs. sunny, day to night or passing clouds, see algorithm in Fig. 1B). Figure 1C presents the external structure and shape of the PCB and completed collar.

When a change in light intensity crosses the amplitude transient threshold levels, the system logs the real-time clock data and transfers SRAM buffered data to the 32kB ferroelectric permanent memory. To avoid high power consumption from the permanent memory, it is only actuated when such transitions that last > 4 s occurs. A miniature magnetic switch mounted on the PCB bestows the possibility of in field activation with a simple 3 magnet swipes performed within 10 s. The redundancy affords the prevention of false activations and battery economy by avoiding actuation of the system prior to its final installed deployment time. The microcontrollers were programmed with a custom host firmware. This configuration allows parameter modification in the module via a RS-232 to USB terminal interface and a simple terminal software on a personal computer.

### Assembling the collar

To assemble the collar, the PCB was first slid into a transparent 2-cm heat-shrink sleeve. A tie-wrap was then slid under the PCB inside the heat-shrink sleeve. Only then was the shrink heated with a heat gun, which fixed the PCB on the tie-wrap. To keep away any water or humidity from the PCB, both ends of the reduced heat-shrink were filled with acetic acid free silicon without touching the PCB itself. Due to the heat-shrink sleeve covering the ambient light sensor, light intensities that are recorded do not represent direct sunlight, but the transparency of the sleeve allowed a reliable proxy.

### Impact of collars on lemmings in captivity

Adult brown lemmings have a minimum weight of ~ 30 g, whereas collared lemmings start at ~ 40 g [20, 21]. The mass of the collar (1.59 g) was ≤ 5% of the body mass of adult lemmings. Keeping tracking devices below a 5% threshold is recommended [22], but could still negatively impact behaviors and vital rates (see [23] for a review; [24, 25]). We evaluated how collars impacted lemmings by comparing body mass changes, a proxy of body condition, and recapture rates, a proxy of survival or behavioral alteration, between lemmings with and without collars. In November 2019, 4 brown lemmings (*Lemmus trimucronatus*) and 2 collared lemmings (*Dicrostonyx groenlandicus*) were held in captivity in Cambridge Bay, Nunavut, Canada. They were provided ad libitum food and water before and during the experiments. For more details about how the lemmings were live-trapped in the field, for the housing conditions and care given to the captive lemmings, see [26].

**Fig. 2:**
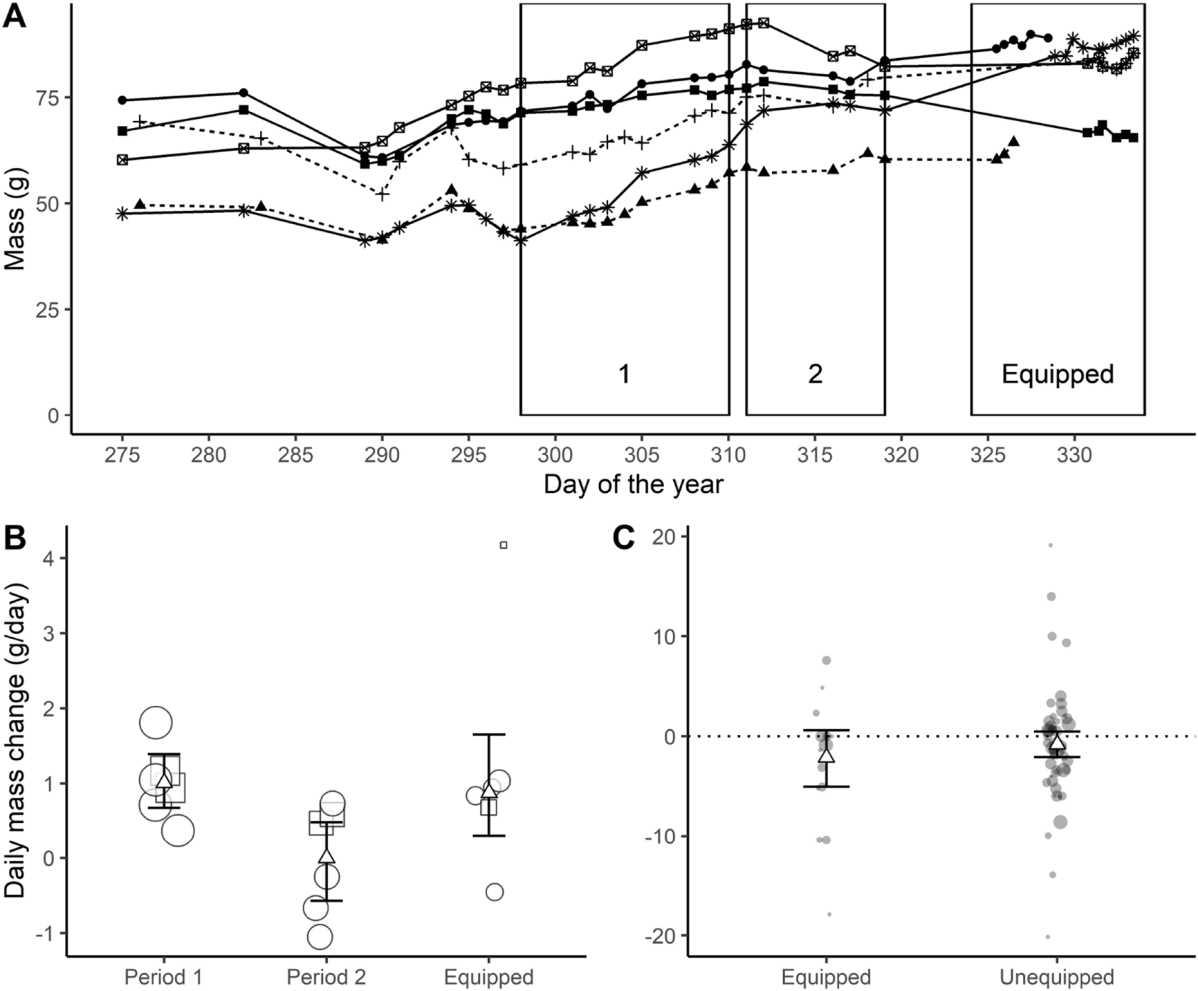
Impact of a photosensitive collar on the daily mass change of captive and wild lemmings. **A**, **B** The masses of six captive lemmings were monitored across 58 days in Cambridge Bay, NU, Canada. Period 1 and 2 are periods when lemmings are not equipped with a 1.5 g dummy collar, contrary to the Equipped period. In each period, daily mass gain was calculated. **A** Body mass of 4 captive brown (full lines) and 2 collared (dotted lines) lemmings. Rectangles delimit different periods. Symbols represent different individuals. **B** Daily mass changes of captive lemmings with 95% CI. The white triangle represents the weighted mean in each period, and squares and circles represent, respectively, brown and collared lemmings. Point size is proportional to the number of days the daily mass change is based on. **C** Field observations of daily mass changes of equipped and unequipped brown lemmings with their 95% CI in the Bylot Island, NU, Canada. White triangles are the weighted mean. Point size is proportional to the number of days the daily mass change is based on, here the time between captures)

The experiment consisted of all lemmings being monitored daily without a collar for several days (lemmings were monitored since August 2019 after their initial capture [26]), and then equipped with a 1.5 g dummy collar between 24 and 108 h (i.e., a tie-wrap with a mass fixed by a heat-shrink). Each individual was kept under observation for the first 15 min and then checked every 2 h for the first 8 h, then every 12 h, to ensure the collars were not causing drastic changes in behavior (e.g., constantly scratching or trying to take off the collar) or choking. The body mass of each lemming was monitored with an electronic scale (± 0.01 g) every day to every week before the collar was installed on it. Once the collar was fit on the lemming, the body mass was measured every day. To determine if collars had an impact on the body conditions of lemmings, we compared the daily mass change of equipped lemmings to their daily mass change before they had the collars on. Two different (non-overlapping) pre-experimental periods of 12 or 8 days were chosen as controls, because lemmings either continuously gained or had a stable mass during these periods (Fig. 2A). We performed a one-sided *t*-test, weighting for the duration of the monitoring in each period, to test the hypothesis that equipped individuals had a lesser daily mass gain than when unequipped.

### Impact of collars on lemmings in the field

We deployed light-sensitive collars on small mammals in three locations of the Canadian Arctic where populations are monitored every year and assessed the impact of collars on body condition and recapture probability. Rodents fitted with collars were brown lemmings (*n* = 5 and 36) and northern collared lemmings (*Dicrostonyx hudsonius*, *n* = 11 and 0) in, respectively, in Cambridge Bay and Bylot Island, Nunavut, whereas Ungava collared lemmings (*n* = 6), an Eastern meadow vole (*Microtus pennsylvanicus*) and a Northern Bog Lemming (*Synaptomys borealis*) were fitted with a collar in Salluit, Quebec. All rodents were monitored at these sites with live-trapping and capture–mark–recapture methods as part of multi-annual surveys. At all sites, trapping grids made of 96 to 144 live-trapping stations, each station being separated by 30 m, and arranged according to a cartesian plane were used (Bylot Island: 3 grids; Cambridge Bay: 4 grids Salluit: 2 grids). Longworth and Little Critter traps were used at all these sites. Capture–mark–recapture methods consisted of opening and baiting traps followed by visits of traps every 12 h until 6 visits were completed. All lemmings captured were marked with a PIT- or ear-tag, weighed and sexed. During live-trapping, adult lemmings with a minimum body mass of 34 g (to ensure that collars accounted ≤ 5% of the total body mass) were fitted with collars. The total number of collars deployed at each site differed due to low lemming densities in both Cambridge Bay and Salluit (< 1 ha^−1^), while lemming densities were high on Bylot Island (15 ha^−1^; unpublished data). All manipulations were approved by the Animal Care Committees of the Canadian Museum of Nature (2018.02.001) and Université Laval (2019-253, VRR-18-050), Parks Canada (SIR-2021-39399), Department of Environment of Nunavut (WL2019-038), Kitikmeot Inuit Association (KTX119N006), and Ministère des Forêts, de la Faune et des Parcs du Québec (SEG 2021-05-31-125-10-S-F).

Recapture probabilities of individuals with and without collars were calculated for each trapping grid. Here, recapture probabilities were calculated as the total number of recaptures across all individuals divided by the total number of captures (i.e., sum of first captures and recaptures). To test if recapture probabilities of equipped individuals were lower than those of unequipped individuals, we used a one-sided t-test with weighted observations to account for the number of deployed collars per grid. This was done to reduce the influence of grids with low sample size on the statistical test.

Using exclusively the data of Bylot Island, where a peak lemming abundance yielded many more captures than at the other sites, we also evaluated the difference in daily mass change between equipped and unequipped lemmings. The relative daily mass changes and 95% confidence intervals (CI) of each group were weighted for the time between captures. We used a one-sided *t*-test weighted for the time between captures to evaluate if daily mass changes of equipped individuals were lesser than those of equipped individuals.

## Results and discussion

The miniature photosensitive collars that we developed provided detailed information about daily routines of lemmings in natural conditions during the Arctic summer. The collars ended up having a mass of 1.59 g (Table 1), which correspond to ≤ 5% of the body mass of all lemmings and were found to have no impact on body mass or recapture rates after being equipped for as long as 2.5 days in the field and 4.5 in captivity days (Fig. 2A). While on the field, we were able to extract data from 13 of the 26 retrieved collars.

**Table 1:** Mass of each component of the photosensitive collar

Element	Mass (mg)
Tie-wrap	280
Printed circuit board	282
Heat-shrink sleeve	330
Caulking	34
Two zinc air batteries	660
Total	1586

### Impact of collars on lemmings in captivity

For the test in captivity, the one-sided t-test showed that equipped lemmings had daily mass changes that were similar to those observed in pre-equipped periods 1 (*p* = 0.39) or 2 (*p* = 0.94) (Fig. 2B). Thus, the dummy collars had negligible or null effect on daily mass change.

### Impact of collars on lemmings in the field

We found no negative effect of the collars on daily mass change of brown lemmings on Bylot Island based on the weighted one-sided *t*-test (*p*-value = 0.21; Fig. 2C). Similarly, a weighted one-sided *t*-test showed that the recapture probabilities of equipped individuals were not lower than those of unequipped individuals (*p*-value = 0.62), even if recapture probabilities varied across sites (Table 2).

**Table 2:** Sample size (*N*) and recapture probabilities of unequipped and equipped small rodents with a photosensitive collar (*R*) in each live-trapping grid of the Canadian Arctic: Bylot Island (NU), Cambridge Bay (NU) and Salluit (QC)

Location	Trapping grid (coordinates*)	*N*_unequipped_	*R*_unequipped_	*N*_equipped_	*R*_equipped_
Salluit	C (62.22°N, 75.62°W)	16	0.56	5	0.60
	L (62.17°N, 75.68°W)	9	0.44	3	0.33
Cambridge Bay	LPH (69.12°N, 105.42°W)	20	0.50	2	0.50
	LPM (69.11°N, 105.42°W)	30	0.33	8	0.13
	OTH (69.10°N, 104.93°W)	4	0.25	3	0.00
	OTM (69.11°N, 104.95°W)	20	0.55	3	0.67
Bylot Island	LG1 (73.16°N, 79.94°W)	183	0.33	10	0.30
	LG2 (73.15°N, 79.97°W)	202	0.47	15	0.47
	LX (73.15°N, 79.94°W)	171	0.35	11	0.73
Weighted average *R*			0.40		0.43

Recapture probabilities are the odds of recapturing a newly released individual. Recapture probability averages are weighted for the number of captures for unequipped individuals, or deployed collars for equipped individuals in the grid

*Coordinates of each trapping grid are presented in degree decimal with the WGS84 geodetic system

Overall, our results are in line with those of previous studies conducted on the impact of radio collars on small rodents that weigh ≤ 5% of the body mass of the host [27, 28]. Indeed, our study confirms negligible, if any, negative impacts on the body mass of lemmings over time and no noticeable change in behavior. Moreover, we further included an analysis of recapture rates, which both considers potential changes in survival and behavior (e.g., trap-shyness), and yielded no difference caused by the collar. No injury or rash was found on the necks of lemmings after collars were removed, which suggests that the material used for the collar (i.e., tie-wrap and heat-shrink) ensured a certain level of comfort. However, the short wearing time (4.5 days in captivity and 2.5 in the wild) prevents us from assessing potential long-term impacts of the collars. We could not conduct the same tests in the field with the other small rodent species, which calls for further assessments. However, most of the literature cited in this article showing weak or no effect of ultra-light collars on small mammals were conducted on voles. Thus, similar results as those observed here for lemmings should apply to other Arctic small rodents that weigh > 30 g.

### Light-sensitive collars to detect circadian rhythms in cryptic species

**Fig. 3:**
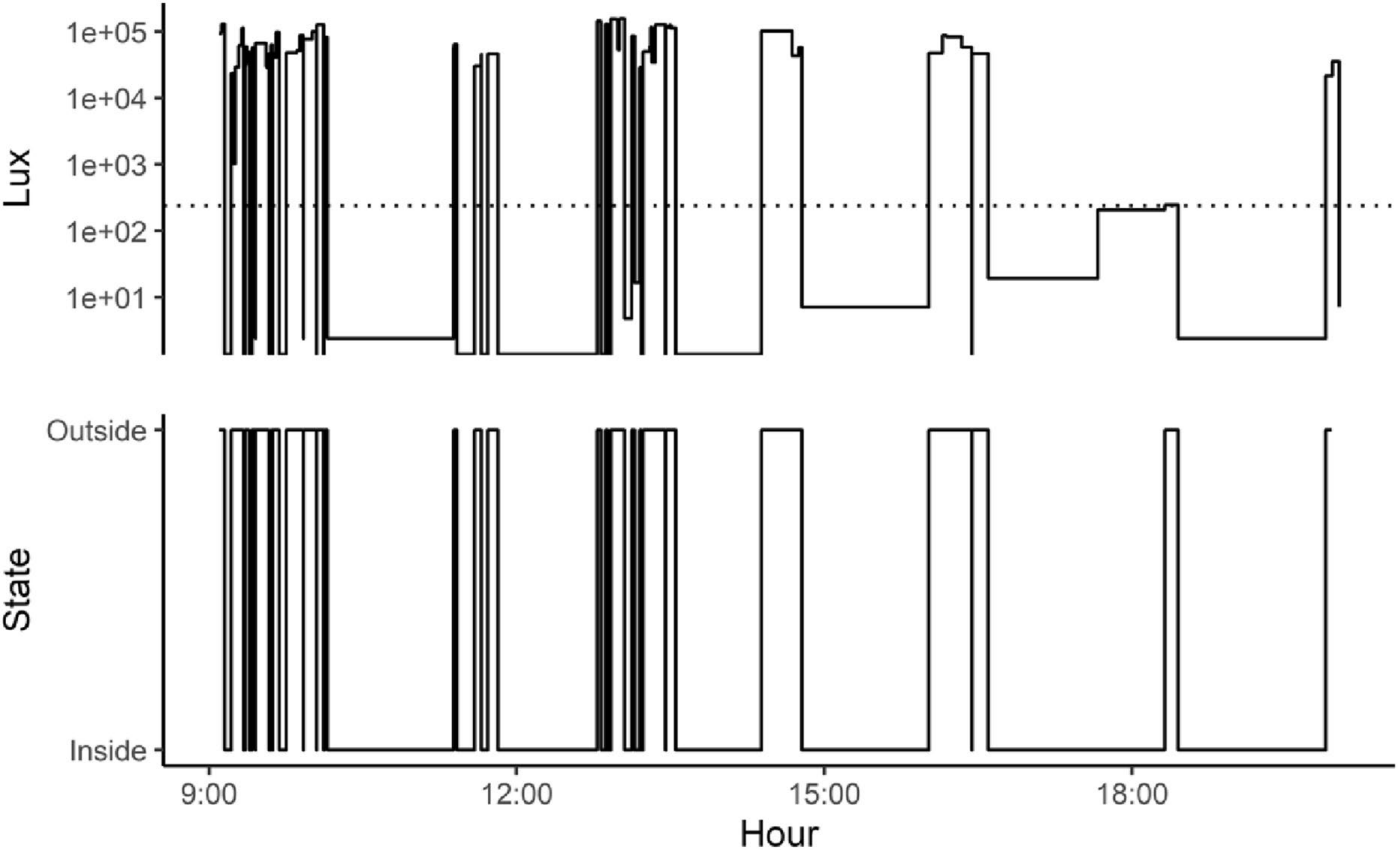
Example of light intensities (lux) recorded by a photosensitive collar equipped on a brown lemming individual on Bylot Island, NU, Canada. Above: continuous light intensity (Lux) on a logarithmic scale over time, with the threshold fixed at 240 lx (dotted line) that separate states of the lemming being inside or outside its burrow. Long periods without changes in lux (flat horizontal lines) indicate no transitions and that the rodent is constantly in darkness. Below: state of lemming being either inside or outside a burrow derived from the recorded light intensity threshold

Light-sensitive collars recorded multiple transitions throughout the days (mean 89.73 per day, range [14, 430]), indicating regular movements inside and outside burrows (Fig. 3). For all collars deployed on lemmings, 95% of transitions were recorded between 5 AM and 22 PM. The absence of transition recorded during the night could either be the result of lemmings staying underground during that period, or that the amplitude in changes of light intensities was too low to be detected by the collars. Although the optical sensor can respond to low light intensities (0.01 lx) the minimum trigger thresholds were likely set too high (i.e., 120–240 lx) preventing the recording of transitions in low-light conditions. A potential solution to this problem may be to reduce such threshold to a value close to 0. Indeed, 0 lx were often observed during daytime and were associated with lemmings being in their burrows. Alternatively, different thresholds could be programmed for daytime and nighttime.

**Fig. 4:**
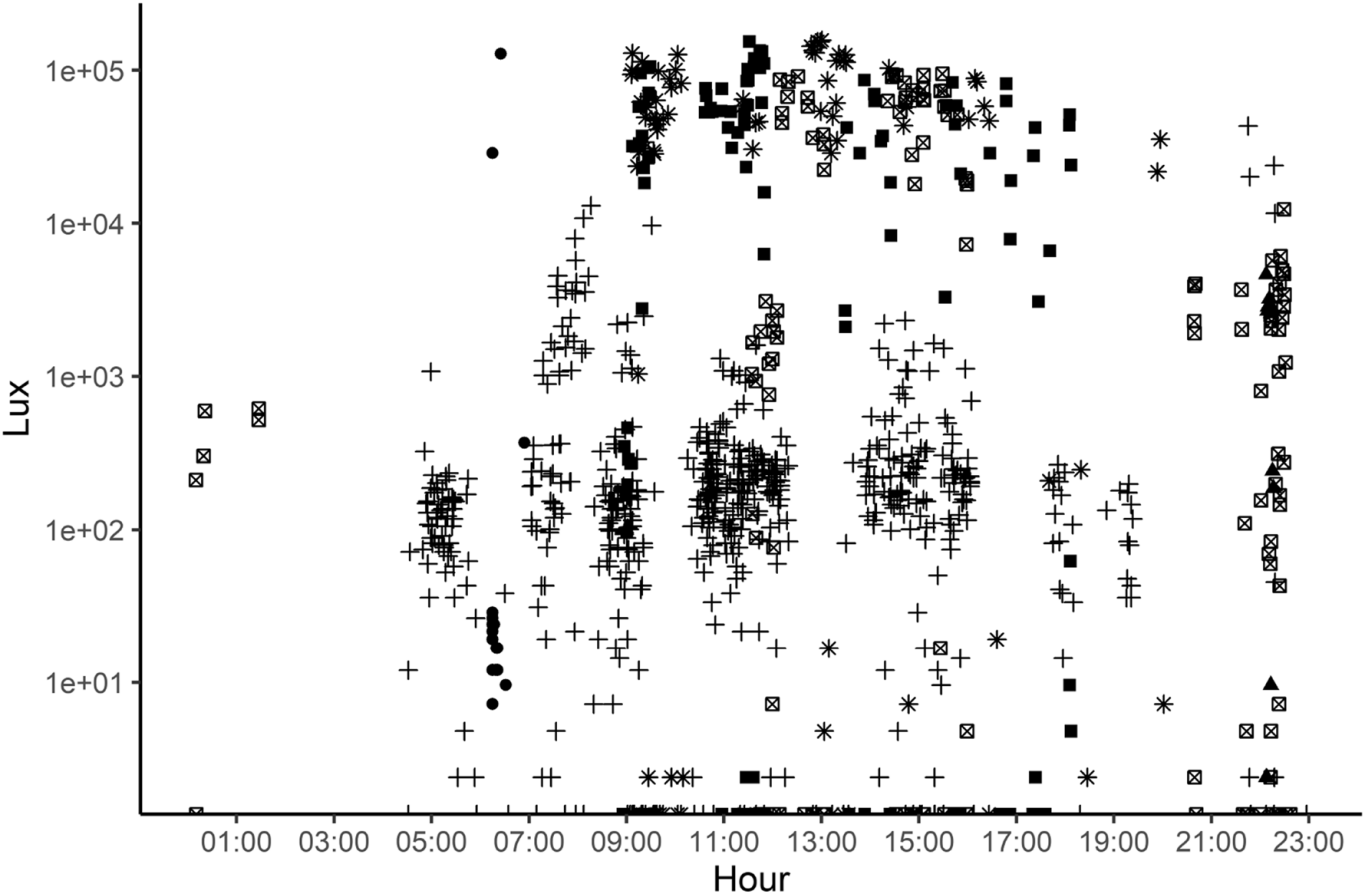
Light intensities (lux) recorded by photosensitive collars equipped on seven brown lemmings, each represented by a different symbol, on Bylot Island, NU, Canada between the 10th and 18th of August 2021. Symbols are used to differentiate individuals

During daylight hours, different individuals simultaneously recorded similar light intensity values, confirming consistency of readings among collars (Fig. 4). Weather patterns, total or partial (e.g., in the shadow of a plant) exposure to the sun, and dirt on the heat-shrink sleeve are all factors that may have contributed to the large variability among recordings taken at the same time of day but on different small rodents. From these values, we derived the state of the lemming (inside or outside its burrow) by discretizing the recorded light intensity by the previously programmed transient amplitude threshold of 240 lx, which was used to record the transition between low and high light intensities. Above it, the lemming was considered outside its burrow. Whether the individuals were at the very entrance of the burrow or further from it is unknown, and this information will influence the degree to which lemming are available to predators [29]. We note in Fig. 4 that the concentration of data points near the transient amplitude threshold could indicate that lemmings avoided being fully exposed and remained in partly shaded areas, such as a burrow entrance, but further tests are needed to clearly distinguish light intensities resulting from weather patterns or behaviour. The results showed a behavioral pattern characterized by continuous bouts of activity outside burrows interrupted by prolonged stays inside burrows. Similar repetitive patterns of activity were also observed in captivity for brown lemmings using running wheels [30] or semi-natural conditions for other fossorial mammals such as bank voles [31] and meadow voles [32] that showed highly fluctuating activity patterns within 24 h periods. High frequency recordings will allow examination of how physiology (e.g., sex, reproductive condition), external parameters (e.g., predation and habitat) and their interactions could affect movements and other behaviors like the use of refuges. Indeed, such parameters have been shown to be important in how fossorial cricetid rodents use burrows as refuges [33].

We found that the batteries, that made up 41% of the total PCB mass, were largely sufficient to record light transitions for at least 72 h in the field and expected to last 20 days from theoretical calculations. Unfortunately, we could not test the longevity of the collars for longer periods in the field due to logistical constraints and short field work season due to the COVID-19 pandemic situation. Additionally, 50% of the retrieved collars contained data. This proportion will be increased by making sturdier connections among all the electronic elements of the collar. Nonetheless, our objectives were fulfilled by developing a fully functional photosensitive collar that can be deployed on rodents of the Arctic tundra with so far no know health risks or impact on behavior. Moreover, this passive recording system that can be set on all Arctic small mammals is one a step further towards revealing some of the most cryptic behaviors with very high details and without observer bias [34].

## Data Availability

The dataset supporting the conclusions of this article is available in the NordicanaD repository in Gauthier, G. 2020. Lemming monitoring on Bylot Island, Nunavut, Canada, v. 1.3 (1994–2019). Nordicana D22, https://doi.org/10.5885/45400AW-9891BD76704C4CE2.
